# Outcomes of Patients with Newly Diagnosed Transplant-Ineligible Multiple Myeloma According to Clinical Trials Enrollment: Experience of a Single Institution

**DOI:** 10.3390/cancers15215261

**Published:** 2023-11-02

**Authors:** Luis Gerardo Rodríguez-Lobato, Natalia Tovar, Anna de Daniel, Carlos Fernández de Larrea, M. Teresa Cibeira, Raquel Jiménez-Segura, David F. Moreno, Aina Oliver-Caldés, Joan Bladé, Laura Rosiñol

**Affiliations:** 1 Amyloidosis and Multiple Myeloma Unit, Department of Hematology, Hospital Clínic of Barcelona, Villarroel 170, 08036 Barcelona, Spain; ntovar@clinic.cat (N.T.); adbisbe@clinic.cat (A.d.D.); cfernan1@clinic.cat (C.F.d.L.); mcibeira@clinic.cat (M.T.C.); raquelhemato@hotmail.com (R.J.-S.); dfmoreno@clinic.cat (D.F.M.); oliver@clinic.cat (A.O.-C.); jblade@clinic.cat (J.B.); 2 Institut d’Investigacions Biomèdiques August Pi i Sunyer (IDIBAPS), 08036 Barcelona, Spain

**Keywords:** multiple myeloma, clinical trial, real world, survival, trial effect, trial enrollment

## Abstract

**Simple Summary:**

Clinical trials (CTs) may not always reflect real-world medical practice, particularly in non-transplant-eligible (NTE) newly diagnosed multiple myeloma (NDMM) patients due to stringent recruitment criteria. Our objective was to assess exclusion rates, reasons, and survival outcomes for NTE NDMM patients in and out of CTs. Roughly 50% did not meet eligibility criteria, mainly due to comorbidities, poor ECOG-PS, or renal failure. CT participation correlated with higher overall response rates (ORRs), improved progression-free survival (PFS) and overall survival (OS), especially in recent years. A slight PFS extension associated with a substantial improvement in OS in CT patients suggested a selection bias. Excluded patients did not experience improved survival, with the median OS remaining unchanged from 2003 to 2017. These findings emphasize that CT participants may not fully represent the wider NDMM population in real-world clinical practice. It is essential to exercise caution when applying CT results to routine care settings, recognizing the limitations of generalization.

**Abstract:**

The proportion of non-transplant-eligible (NTE) newly diagnosed multiple myeloma (NDMM) patients excluded from clinical trials (CTs) and their prognosis is unknown. CT results may not be generalizable to real-world practice due to strict recruitment criteria. We analyzed causes of NTE-NDMM patient exclusion form CTs and their outcomes. A total of 211 NTE-NDMM patients were included. They were divided into three periods: 2003–2007, 2008–2012, and 2013–2017. Overall, 50% received non-trial treatment (NCT), while 50% participated in a CT (20% control group (CG) and 30% experimental group (EG)). Main causes for exclusion from CTs were comorbidities, ECOG > 2, and renal insufficiency. In the first two periods, the CR rate was similar regardless of treatment type, but in the last period, the EG group showed improved CR. Median PFS was similar in the first two periods, with a benefit seen only in the EG in the last period. The median OS was significantly longer in CT-included patients compared to NCT group in the last two periods. Conclusions: The presence of comorbidities and worsened ECOG were the main reasons for CT exclusion. Patients included in CTs had a longer OS than NCT. This OS benefit may be influenced by a selection bias, making it challenging to generalize CT results to real clinical practice.

## 1. Introduction

Multiple myeloma (MM) predominantly affects elderly people [1,2,3]. The prognosis of the disease has improved with the introduction of several novel therapies [3,4,5,6,7,8,9]. However, MM remains a disease associated with high morbidity and mortality [10]. Front-line therapy in non-transplant-eligible (NTE) newly diagnosed MM (NDMM) has significantly evolved in recent years [11]. The armamentarium for this population of patient has evolved from monotherapy to tripe/quadruplet regimens with unprecedented improvements in survival outcomes [10].

Real-life estimates have shown that a low percentage of patients are included in CTs (2–11%). This is mainly due to structural (CTs availability), clinical (eligibility assessment), physician (benefit–risk analysis) and patient (personal decision) obstacles, highlighting that structural and clinical barriers are the main reasons [12,13].

Participation in CTs has benefits and inconveniences. Firstly, the inclusion allows the patient to access the newest treatments before they become commonly available and secondly receiving standardized and comprehensive medical care from an experimented research team. On the other hand, they face the possibility of unknown adverse events, delayed treatment initiation for the screening phase, and consuming more time and attention than outside of the CT [14], even if they are randomized to the control group. It is broadly believed that the benefits of a CT may be due to the so-called “trial effect” or “inclusion benefit.” The underlying sources of the “trial effect” could be explained by several reasons: experimental treatment effect (experimental treatment is superior to standard treatment), protocol effect (strict adherence to treatment regimens and procedures), care effect (incidental aspects of care not considered by protocol effect), Hawthorne effect (changes in patient or physician behavior after close observation), placebo effect, and selection bias (healthier patients with fewer comorbidities) [15,16,17]. Unfortunately, determining the role that each of these effects plays in the outcomes of CTs is a major challenge, and some approximations have already been made with contradictory results [15,18,19].

There are limited data comparing the outcomes of MM patients who participate in CTs and those who do not. Registry studies mostly analyze whether or not patients could potentially be enrolled in a CT, applying the standard inclusion and exclusion criteria of various phase III CTs [20,21,22,23,24,25], making it hard to draw solid conclusions from this approach.

Randomized CTs are the “gold standard” of evidence as they reduce selection bias and confounders [26]. Researchers strive for internal validity by establishing replicable and clear inclusion criteria. However, this approach presents limitations concerning external validity, as it may hinder the generalizability of findings to real-world practice, particularly when there are disparities in the baseline characteristics of patients, such as age, comorbidities, and performance status, and other temporal, ethnic, socioeconomic, and geographic factors exist [27,28,29,30]. It has recently been shown that some real-world efficacy data are comparable with CTs outcomes in MM [31]. However, some patients may not be suitable for new treatment combinations due to their advanced age, multicomorbidity or severely impaired functional status, so the extrapolation of the results of this population is unfeasible.

This study was aimed to determine systematically the proportion of NTE NDMM patients excluded from CTs and to identify the reasons behind their exclusion at a single tertiary center in Spain with expertise in MM and a long-term tradition in CT participation. Additionally, it sought to analyze the survival outcomes of patients who were included in a CT, both in the experimental and control groups, in comparison to those who were not included.

## 2. Materials and Methods

### 2.1. Study Design and Population

A retrospective observational study was performed systematically in all patients with NTE NDMM (*N* = 583) treated at Hospital Clínic of Barcelona between January 2003 and December 2017. Our institution serves patients from a specific section of Barcelona and Catalonia. All individuals diagnosed at our institution with myeloma or referred from community hospitals undergo an evaluation to assess their suitability for participation in CTs. The two hundred sixty-two patients, who were candidates to autologous stem cell transplantation (ASCT) were excluded from this study, and 110 NTE patients were also excluded, as there was no first-line CT available at our medical center at the time of diagnosis. The final study population was comprised by 211 patients, 105 of which were enrolled and 106 were not (2003–2007: 35 (60%) patients; 2008–2012: 37 (52%) patients; 2013–2017: 34 (42%) patients) (Figure S1). All patients had symptomatic MM. The last update of the database was made in May 2022. The CTs available at our center during each year analyzed are summarized in Table 1. Patient recruitment was based on the inclusion and exclusion criteria of each CT. Patients were categorized into three calendar periods (2003–2007, 2008–2012, and 2013–2017). This study was approved by the Hospital Clínic of Barcelona Institutional Review Board, and was in accordance with the Declaration of Helsinki.

### 2.2. Outcomes of Interest

Regarding response assessments, overall response rate (ORR), complete response (CR), partial response (PR), stable disease (SD) and progressive disease were analyzed. Treatment response was assessed by different criteria depending on the year of diagnosis [32,33,34]. The ORR was defined as the proportion of patients who achieved at least a partial response. In terms of survival, progression-free survival (PFS) was defined as time from diagnosis of symptomatic MM to death or progression. Overall survival (OS) was defined as time from diagnosis to death.

### 2.3. Statistical Analysis

Quantitative variables were expressed as median and interquartile range, meanwhile, categorical variables were expressed as proportions (number and percentage). Chi-square test and Fisher’s test were used to compare categorical variables, as well as t-test or Wilcoxon rank-sum test for continuous variables. Univariate and multivariate logistic regression models were built to identify factors associated with response. The starting point for time-to-event analysis was the diagnosis of myeloma. CT enrollment was analyzed as a time-varying covariate. Multivariate analyses were performed using Cox proportional hazard regression with time-dependent covariates and the backward stepwise method to identify factors associated with PFS and OS. Mantel–Byar test was used for survival analysis [35]. All analyses were evaluated using a two-tailed *p* value with an alpha level of 0.05. The Bonferroni correction was calculated to adjust for multiple comparisons. Analyzes were performed using RStudio 2023.03.1 and GraphPad Prism 8.2.1.

## 3. Results

### 3.1. Clinical Trial Enrollment

Of the 211 NTE NDMM patients included in this study, 105 patients (49.8%) were successfully enrolled in a CT. Most patients (88.6%) were included in phase III CTs. The number of patients enrolled in CTs increased over time, from 40% in 2003–2007 to 58% in the most recent period (Figure S2). Regarding the patients included in CT, 43 (41%) received a conventional treatment; control group (CG) and 62 (59%) received an experimental treatment experimental group (EG).

Regarding the 106 patients (50.2%) who were not included in CTs (NCT group), the main reasons for their exclusion were comorbidities (40.6%), ECOG-performance status (PS) >2 (30.2%), renal failure (eGFR <45 mL/min/1.73 m^2^ or renal replacement therapy) (14.3%), very advanced age (12.3%; median 90 years (range 85–92)), patient refusal (10.4%), urgent need for treatment initiation (9.4%), and non-measurable disease (3.8%) (Table 2). Previous malignancies (19/43 (44.2%)) and hepatitis C virus infection (5/43 (11.7%)) were the main excluding comorbidities. Table 2 summarizes why patients were excluded from CTs, and Table 3 summarizes the list of comorbidities in patients not included in CTs.

### 3.2. Patient Baseline Characteristics

Table 4 summarizes the baseline features of the 211 patients included in this analysis. Patients enrolled in CTs were younger (median age CG: 72 years; EG: 70 years; NCT: 78 years; *p* < 0.001), with a lower proportion of patients >75 years of age (CG: 32.6%, EG: 27.4%, NCT: 60.4%; *p* < 0.001), had a better renal function (creatinine >1.5 mg/dL; CG: 11.6%, EG: 12.9%, NCT: 29.2%; *p* = 0.010), lower lactate dehydrogenase elevation (LDH) (CG: 11.6%, EG: 10.2%, NCT: 25.5%; *p* = 0.024), and lower ECOG-PS > 2 (CG: 0%; EG: 6.7%; NCT: 30.8%; *p* < 0.001). No statistically significant differences were observed between the groups in relation to immunological subtype, bone marrow plasma cell infiltration, CRAB symptoms, and the International Staging System (ISS).

### 3.3. First-Line Treatment Outcomes

The median follow-up duration of the entire population included in the analysis was 8.6 years (interquartile range (IQR) 6.8–13.8), being 2.5 years (IQR 0.8–4.5) for the non-CT patients and 8.5 years (IQR 6.7–13.8) for the patients included in CTs. First-line treatment combinations for the patients not included in CTs were chemotherapy-based regimens in 79.2% and 18.9% proteasome inhibitors (PI)-based regimens. Patients included in CTs received PI-based combinations (CG: 44%, and EG: 42%) and PI plus immunomodulatory drug (IMiD)-based combinations (CG: 14% and EG: 15%). Regarding monoclonal antibodies-based schemes, the EG (18%) was the only group that received them (Table 4). The median number of days elapsed between the diagnosis of symptomatic MM and the start of treatment was longer in patients included in a CT (28 days (IQR 16–41) vs. 16.5 days (IQR 7–33); *p* = 0.002; Figure S3).

### 3.4. Responses

Concerning responses after first-line treatment, the ORR was higher in the CT group (CG: 74% and EG: 76%) compared with the NCT group (43%); *p* < 0.001. Similarly, the depth of response (CR) was also superior in the CT group (CG and EG: 16% vs. NCT: 3%; *p* < 0.001) (Table 5). Regardless of the type of treatment received, ORR and the CR rate were similar in the first two analyzed periods. However, in the last period (2013–2017) the EG had a higher ORR and the CR rates (NCT: 41%, CG: 74%, EG: 84%, and NCT: 3%, CG: 17%, EG: 28%, respectively; *p* = 0.002) (Table 5). When results were adjusted for covariates in a multivariable logistic regression model, the response benefit was found among those enrolled (OR 3.39; *p* = 0.0002) vs. not enrolled in CT, while the presence of renal failure (OR 0.42; *p* = 0.021) and age >75 years (OR 0.43; *p* = 0.008) were variables associated with worse ORR (Table 6). 

### 3.5. Progression-Free Survival

**Figure 1:**
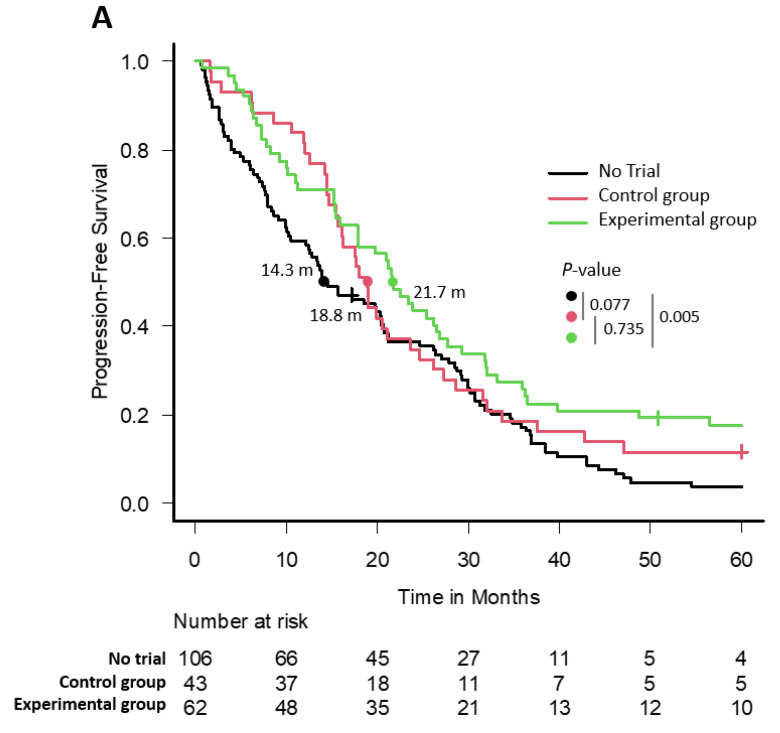

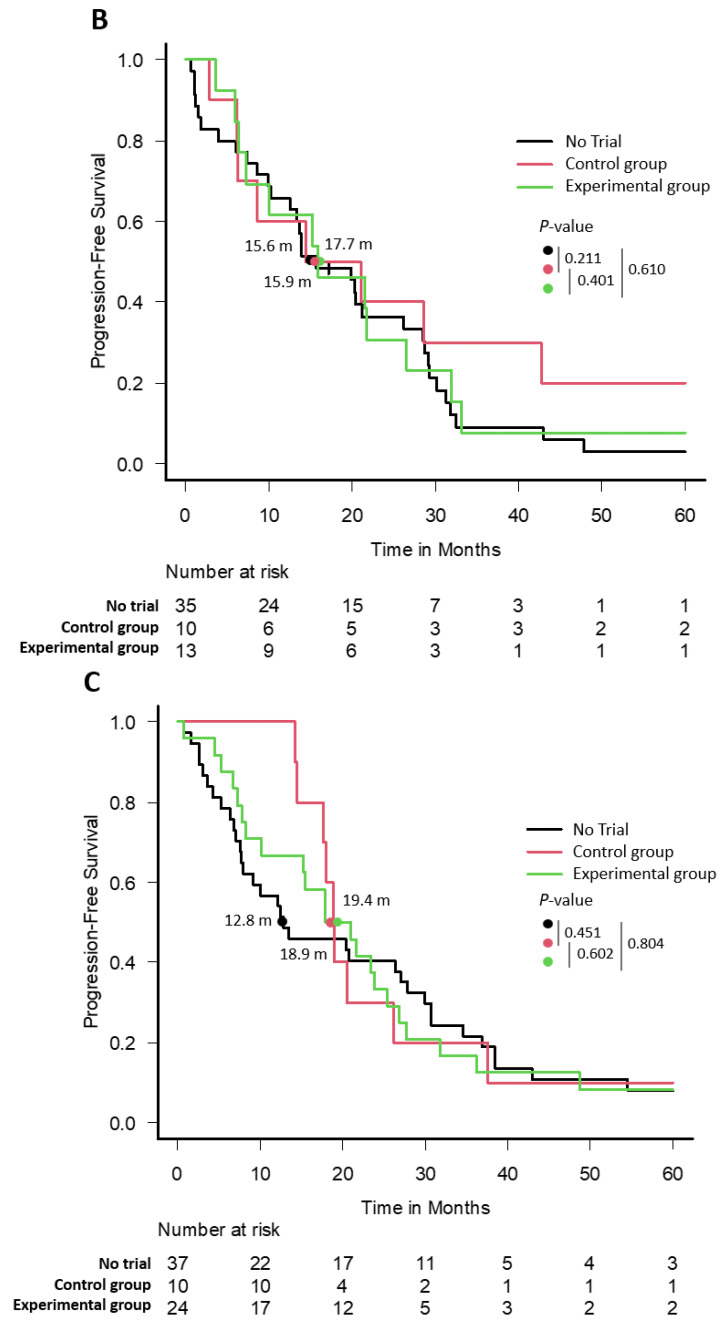

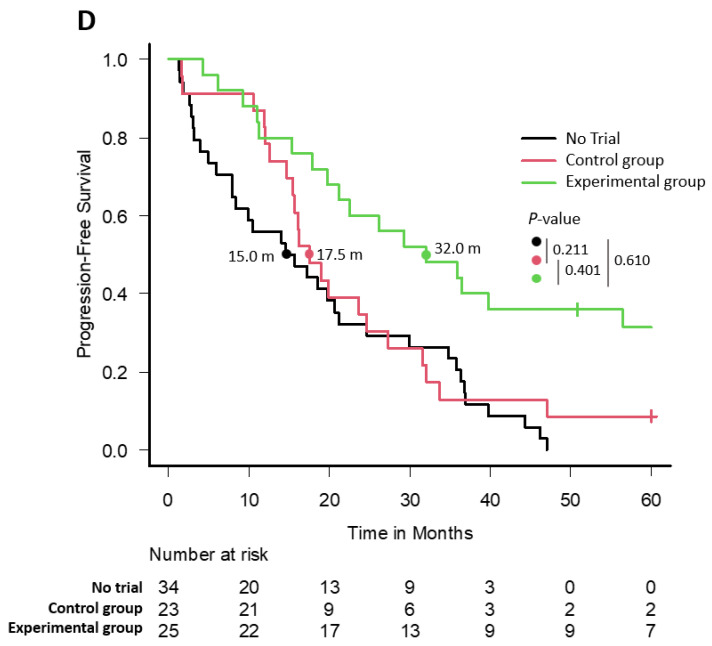
PFS plot in the entire series (**A**), 2003–2007 (**B**), 2008–2012 (**C**), and 2013–2017 (**D**).

In terms of PFS, there were a total of 202 events, with nine patients not experiencing progression or death. The median PFS of the EG patients was slightly better than that of the not enrolled patients: EG: 21.7 months (95% confidence interval (CI) 15.9–26.8) vs. NCT: 14.5 months (95% CI 10.4–20.1), *p* = 0.005; Hazard Ratio (HR) 0.63 (95% CI 0.45–0.87), *p* = 0.006) for the entire time period. However, there were not statistically significant differences between NCT vs. CG and CG vs. EG groups (Figure 1A). In the first two calendar periods analyzed (2003–2007 and 2008–2012) no improvements in PFS were observed in those patients included in CTs regardless of whether they were randomized to the CG or the EG (mPFS: CG 17.7 months vs. EG 15.9 months vs. NCT 15.6 months; *p* = 0.685, and CG 18.9 months vs. EG 19.4 months vs. NCT 12.8 months; *p* = 0.793, respectively) (Figure 1B,C). The isolated benefit was observed in patients who were enrolled in CTs and received the experimental treatment during the last 5 years analyzed (2013–2017) (32 months (95% CI 19.7–56.5)) compared with both the CG (17.5 months (95% CI 14.7–24.6); HR 0.54 (95% CI 0.29–1.00); *p* = 0.048) and the NCT group (15.1 months (95% CI 7.8–21.1); HR 0.35 (0.19–0.65); *p* < 0.001) (Figure 1D). The 3-year PFS in the last period (2013–2017) analyzed rate was 20.6% (95% CI 9.1–35.3) for NCT patients, 13.0% (95% CI 3.3–29.7) for the CG, and 44% (95% CI 24.5–61.9) for patients randomized to the EG (Table S1). The multivariable Cox proportional hazard model that accounted for CT enrollment as a time-dependent covariate showed that the participation in CTs was a protective factor (HR 0.70; *p* = 0.019), and conversely, the presence of hypercalcemia (HR 1.71; *p* = 0.029), renal failure (HR 1.61; *p* = 0.011), and plasmacytoma (HR 1.72; *p* = 0.004) were considered risk factors for a worse prognosis (Table 7).

### 3.6. Overall Survival

**Figure 2:**
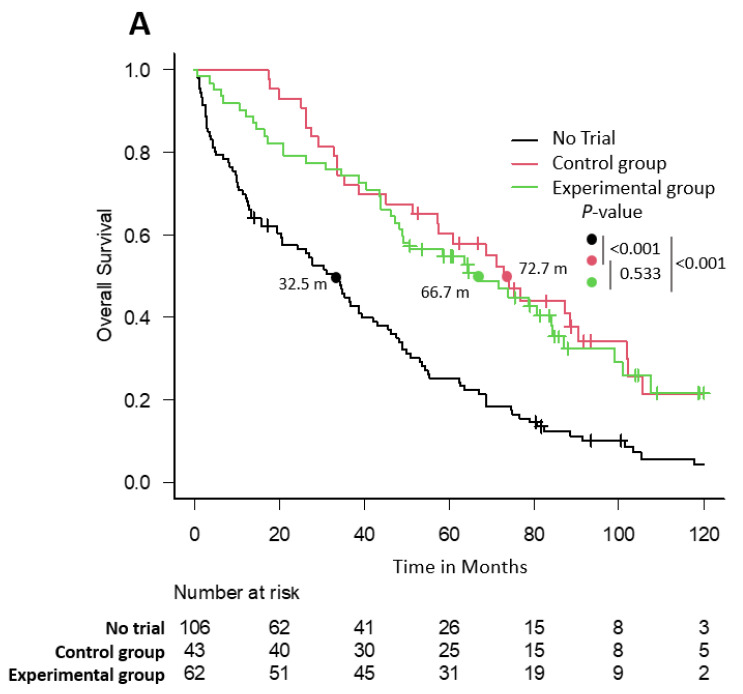

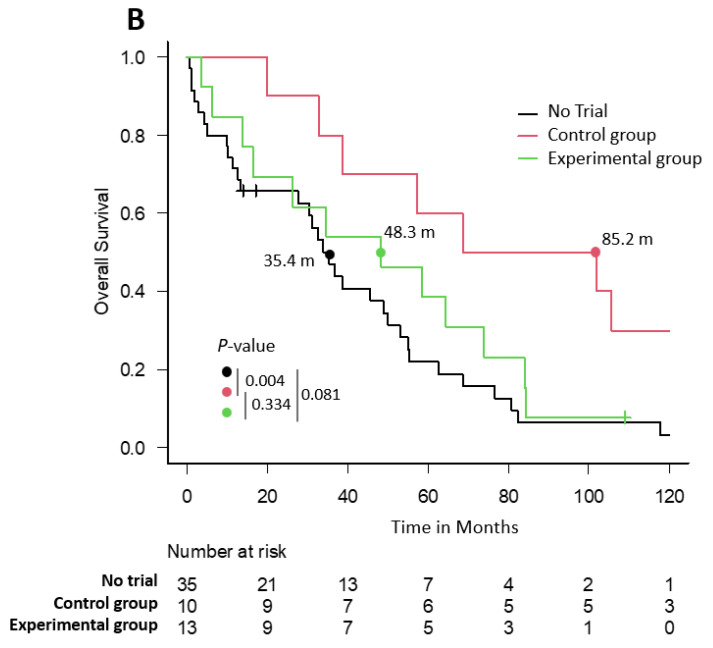

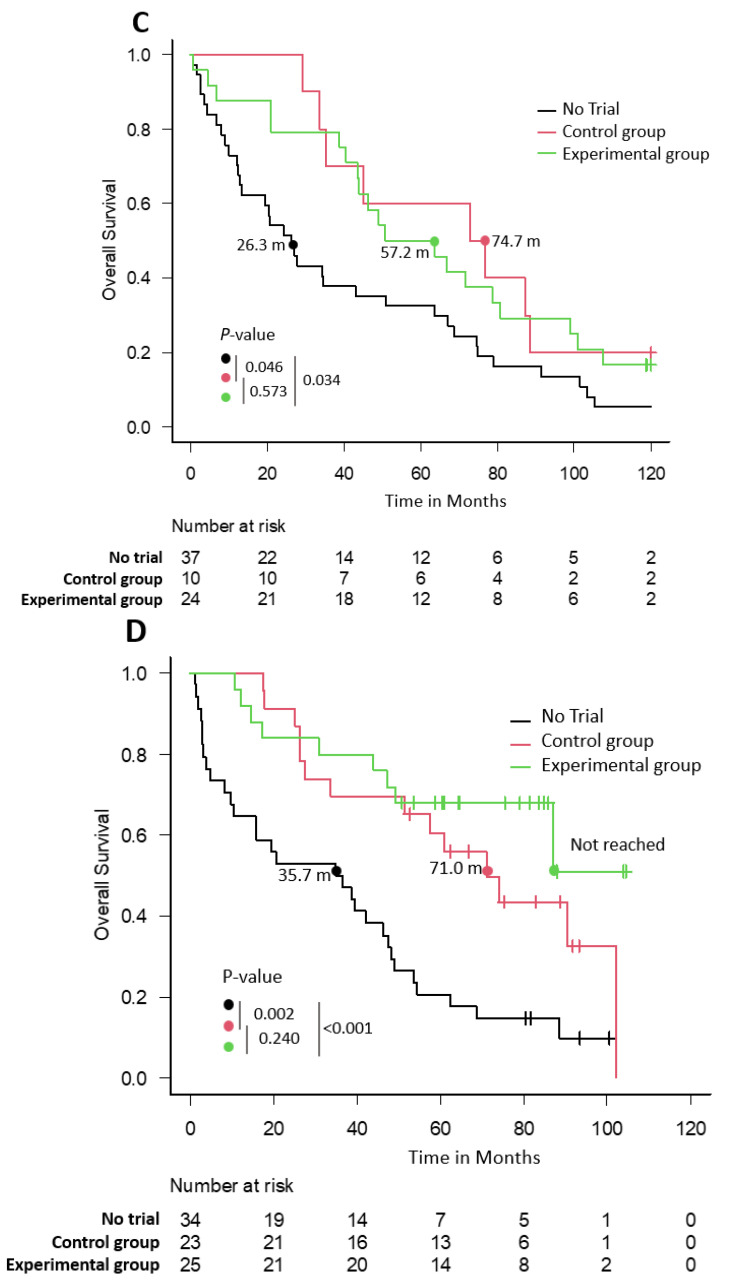
OS plot in the entire series (**A**), 2003–2007 (**B**), 2008–2012 (**C**), and 2013–2017 (**D**).

Regarding OS for the entire series, there were 171 events observed, and 40 patients are still alive. The median OS of the trial-enrolled patients (CG and EG) was markedly longer compared with the trial-ineligible patients: CG, 72.7 months (95% CI 51.3–90.5); EG, 66.7 months (95% CI 47.2–84.3) vs. NCT, 32.5 months (95% CI 20.4–39.4); *p* < 0.001 (Figure 2A). This benefit was observed in all the periods analyzed (Figure 2B,C), and particularly in the last period (2013–2017): CG, 71 months (95% CI 33.6-not reached (NR)) and EG NR (95% CI 49.3-NR) vs. NCT, 35.7 months (95% CI 9.7–47.6); *p* < 0.001; CG vs. NCT with a HR of 0.37 (95% CI 0.19–0.72); *p* = 0.003; EG vs. NCT with a HR of 0.26 (95% CI 0.12–0.54); *p* < 0.001. There were no statistical differences between the CG and the EG in any period analyzed (Figure 2B–D) in terms of median OS. The 5-year OS rate in the most recent period analyzed (2013–2017) was 20.6% (95% CI 9.1–35.3) for NCT patients, 60.6% (95% CI 37.8–77.2) for the CG, and 68% (95% CI 46.1–82.5) for patients randomized to the EG (Table S2). The multivariate Cox regression with a time-dependent covariate model for OS showed that enrollment in CTs maintained its protective effect (HR 0.60; *p* = 0.003). Baseline variables associated with worse prognosis were age >75 years (HR 1.69; *p* = 0.003), renal failure (HR 1.60; *p* = 0.040), the presence of plasmacytomas (HR 2.28; *p* < 0.001), ECOG PS >2 (HR 1.62; *p* = 0.041), and ISS of III (HR 2.07; *p* = 0.005) (Table 8).

### 3.7. Outcomes after Front-Line Therapy

**Figure 3:**
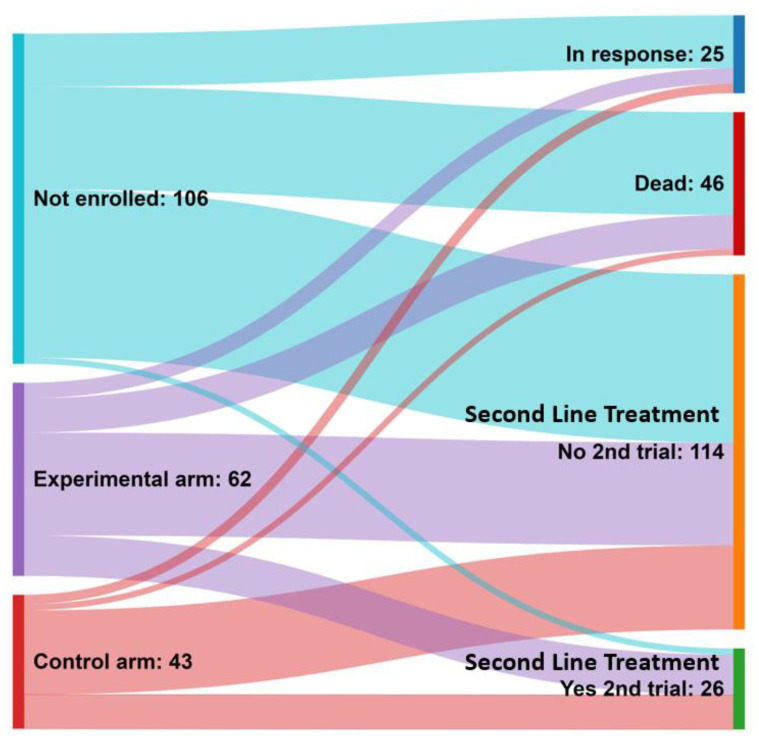
Sankey plot depicting the type of treatment received as second-line therapy in relapsing patients.

Finally, we aimed to determine the attrition rate after first-line treatment and assess patients’ inclusion in a second-line CT. Additionally, we analyzed the total number of lines of therapy (LOTs) received by patients. Figure 3 illustrates that among 211 patients, 12% (25/211) remained alive and progression-free, having received only upfront therapy. Unfortunately, 22% (46/211) of patients died and were unable to receive second-line treatment, with 72% (33/46) belonging to the NCT group. Among the relapsed patients who were still alive (*n* = 139), 81.2% (113/139) received standard second-line treatment outside of a CT, while 18.8% (26/139) were eligible for second-line CT. Notably, 92% (24/26) of these patients had previously participated in a CT during front-line therapy, with 50% (13/26) receiving the experimental treatment and 42% (11/26) receiving the control treatment. In terms of the number of LOTs, a significantly lower proportion of patients in the NCT group (25.5%) received more than two LOTs compared to those included in a CT (48.6%; *p* < 0.001) (Table 4).

## 4. Discussion

This real-world data analysis focused on NTE patients with NDMM showed that half of them did not meet the eligibility criteria for CTs. The main exclusion criteria were the presence of comorbidities, poor PS, and renal failure. Overall, in terms of efficacy, patients included in CTs exhibited deeper responses and longer survival outcomes. However, a significant improvement in PFS was only observed during the most recent period analyzed (2013–2017).

There is limited data on MM patient participation rates in CTs and particularly the reasons for their non-participation. Regarding clinical barriers (inclusion/exclusion criteria), around 40% (range, 22–72%) of patients with MM do not meet phase III trial criteria [20,25,36,37,38]. In our study, 50% of patients were excluded for not meeting the inclusion/exclusion criteria, with malignancies, a low ECOG-PS, and renal failure constituting the primary reasons for exclusion. These results are consistent with findings from registry studies [20,25]. The absence of measurable disease is another common exclusion criterion, affecting up to 25% of patients [20]. Patient refusal accounts for less than 10% of CT barriers. Some uncertainties surrounding trial participation include potential toxicity, randomization procedure, the experimental nature of the research, the need for closer follow-up, caregiver availability, physician–patient relationship, and patient’s age [39]. Waiting time for treatment can also impact trial participation. In this sense, we found that the time between diagnosis and the start of treatment was significantly longer in patients included in CT (28 vs. 16.5 days). This fact is an important selection bias as patients who need urgent therapy are not considered for inclusion in CT.

In the myeloma field, most registry- and population-based studies highlight that patients eligible for CTs have a better prognosis regarding PFS and OS [20,21,22,23,24,25,37,38]. Despite this, some authors have questioned the existence of a “trial effect” in cancer patients [15,19,40]. We conducted a study analyzing real-world patients included in a trial, observing significant improvements in the quality of the responses obtained and longer PFS and OS. Multivariate analyses confirmed the beneficial effect of inclusion in CTs. However, we noted a poor correlation between PFS and OS among patients enrolled in CTs during the first 10 years analyzed. Specifically, there was no observed benefit in terms of PFS in patients who participated in CTs compared to non-participating patients from 2003 to 2012. However, although there was no clear benefit observed in PFS, patients enrolled in CTs demonstrated a significant OS advantage. This discrepancy can potentially be attributed to the fact that CT participants generally possess more favorable baseline characteristics, such as younger age, better functional status, and renal function. As a result, these patients are more likely to receive multiple LOTs following the first relapse and have access to novel treatment options throughout the course of their myeloma, ultimately leading to extended OS. Notably, our findings underscore that patients who were not enrolled in CTs had limited opportunities to undergo more than two LOTs compared to those who participated in CTs (25.5% vs. 48.6%).

The survival rate of patients with MM included in CTs improved significantly in this study, especially between 2013 and 2017. This can be attributed to the use of new drugs and combinations, especially anti-CD38 and SLAMF7 monoclonal antibodies and carfilzomib. Patients who were enrolled in a trial during this period had a median OS of 87 months, which is a remarkable milestone. The ALCYONE trial reported a median OS of 82.7 months for the experimental group [41], while the experimental group in the MAIA trial had a 60-month OS rate of 66.6% [42].

A significant finding of this study is that patients who were excluded from CTs did not experience improved survival rates during the analyzed periods. The median OS was 35 months from 2002 to 2007, compared to 36 months from 2013 to 2017. This finding underscores that patients who are not included in CTs tend to have worse baseline characteristics, which in turn leads to less suitable treatment approaches and poorer prognoses. This population is not representative of the patients typically included in CTs, making it challenging to generalize or extrapolate trial outcomes to this particular patient demographic.

This study has several limitations, including its retrospective nature and single-center setting. We did not measure parallel aspects such as patient satisfaction, compliance, or quality of life. This study cannot definitively determine the role played by each factor that contributes to the “trial effect”. There is no optimal way to dissect it, but some hypothetical recommendations have already been postulated [15]. Another potential bias could arise from the heterogeneity in treatments administered across different groups and periods under analysis. However, it is important to emphasize that the primary aim of this study is not to determine the effectiveness of individual treatment combinations but rather to evaluate the outcomes associated with participation or non-participation in CTs. The strengths include the whole patient population from a highly specialized tertiary MM institution, high-quality data collection that includes clinical and laboratory characteristics often lacking in international registry studies, and close and long-term patient follow-up by an experienced team.

Encouraging the participation of patients with MM in well-designed CTs that analyze promising molecules or products should be a top priority [43,44]. To achieve this, CT availability should be increased and a greater effort made to broaden the eligibility criteria. This would benefit more patients and reduce the gap between trial and real-world populations. However, it should not be overlooked that, despite this, there are still patients with MM who will be too old, with so severe comorbidities and/or such deteriorated general condition that they will be unable to receive novel therapies or combinations and will have an ominous prognosis. In these cases, we must offer these patients relief and comfort through appropriate palliative management.

## 5. Conclusions

In summary, this study provides valuable insights into the actual rate of CT inclusion among patients with NDMM who are NTE at a tertiary academic center. Poor ECOG-PS, renal failure, and previous malignancies were the primary factors leading to non-enrollment in CTs. In our series, participation in CTs was associated with higher ORR, as well as improved PFS and OS. Although there was a slight extension in PFS, the substantial benefit observed in OS among patients included in CTs might indicate the presence of selection bias, such as need for immediate therapy, high-risk cytogenetic alterations, or frailty. Patients enrolled in CTs do not fully represent the broader population in real-world clinical practice, so being aware of this is of vital importance when extrapolating CT results to routine care practice.

## Figures and Tables

**Table 1:** Available clinical trials and number of patients included.

Clinical Trial	Phase	Drug or Combination	Patients (%)	Year
THAL-003	III	TD vs. D	14 (13.3)	2003
VISTA	III	VMP vs. MP	3 (2.9)	2005
GEM05MAS65	III	VMP vs. VTP	2 (1.8)	2006
CC-5013-015	III	Rd vs. D	7 (6.7)	2007
REN-VEL	III	VD	7 (6.7)	2010
GEM2010mas65	III	VMP/RD	15 (14.3)	2011
CNTO 328 MMY 2001	II	CNTO + VMP	8 (7.6)	2011
C16006	I/II	Ixazomib-MP	3 (2.9)	2012
CA204006	III	Elotuzumab +/− Rd	8 (7.6)	2013
Clarion-ONYX 2012-005	III	KMP vs. VMP	12 (11.4)	2013
54767414MMY1001	Ib	Dara + backbone regimen	1 (1)	2014
Alcyone 54767414MMY3007	III	Dara-VMP vs. VMP	11 (10.5)	2015
GEM-Claridex	III	RD+/− Clarithromycine	14 (13.3)	2016

CNTO: Anti-IL-6 monoclonal antibody; D/d: dexamethasone; Dara: daratumumab; K: carfilzomib; M: melphalan; P: prednisone; R: lenalidomide; T: thalidomide; V: bortezomib.

**Table 2:** Reasons for non-inclusion in clinical trials.

Characteristic	Number of Patients (%) *
Comorbidities	43 (40.6)
ECOG-PS > 2	32 (30.2)
Renal failure **	15 (14.3)
Advanced age	13 (12.3)
Patient refusal	11 (10.4)
Urgent start of treatment	10 (9.4)
Non-measurable disease	4 (3.8)

ECOG-PS: Eastern Cooperative Oncology Group Performance Status. * The percentage of patients is >100, because there were patients who had more than one reason for not being included in a CT. ** eGFR < 45 mL/min/1.73 m^2^ or renal replacement therapy is contingent on the specific criteria outlined within each clinical trial.

**Table 3:** Comorbidities in patients not included in clinical trials.

Comorbidities	Number of Patients *N* = 43 (%)
Previous malignancy *	19 (44.2)
Hepatitis C virus infection	5 (11.7)
Cognitive impairment	2 (4.7)
Ischemic heart disease	2 (4.7)
Parkinson’s disease	2 (4.7)
Renal transplantation	2 (4.7)
Heart failure	1 (2.3)
Severe chronic pneumopathy	1 (2.3)
Cerebrovascular disease	1 (2.3)
Infectious disease (endocarditis)	1 (2.3)
Schizophrenia	1 (2.3)
Major depression	1 (2.3)
Alcohol-use disorder	1 (2.3)
Liver transplantation	1 (2.3)
Active bleeding (angiodysplasias in ileum)	1 (2.3)
Severe myopathy	1 (2.3)
Polymyalgia rheumatica	1 (2.3)

* Prostate (*N* = 5), breast (*N* = 4), gastrointestinal (*N* = 4), lung (*N* = 2), head and neck (*N* = 1), melanoma (*N* = 1), angiosarcoma (*N* = 1), essential thrombocythemia (*N* = 1).

**Table 4:** Main patient characteristics.

Characteristics	Not Enrolled Group *N* = 106	CT group		*p*-Value NCT vs. CG vs. EG
		Control Group *N* = 43	Experimental Group *N* = 62	
Age, median (range)	78 (65–92)	72 (61–86)	70 (60–84)	<0.001
>75 years, N (%)	64 (60.4)	14 (32.6)	17 (27.4)	<0.001
Sex, male, N (%)	45 (42.5)	20 (46.5)	34 (54.8)	0.216
Immunological subtype, N (%)				0.583
IgG	59 (55.7)	21 (48.8)	31 (50.0)	
IgA	28 (26.4)	14 (32.6)	24 (38.7)	
Bence Jones	10 (9.4)	7 (16.3)	4 (6.5)	
IgD	4 (3.8)	0 (0.0)	2 (3.2)	
IgM	2 (1.9)	0 (0.0)	0 (0.0)	
Non-secretory	1 (0.9)	0 (0.0)	1 (1.6)	
Biclonal	2 (1.9)	1 (2.3)	0 (0.0)	
Light chain isotype, N (%)				0.591
Kappa	59 (55.6)	29 (67.4)	35 (56.4)	
Lambda	44 (41.5)	13 (30.2)	27 (43.5)	
Biclonal	3 (2.8)	1 (2.3)	0 (0.0)	
Bone marrow plasma cells, median (IQR)	32 (19–50)	33 (20–54)	37 (19–55)	0.622
CRAB symptoms, N (%)				
Calcium >11.0 mg/dL	13 (12.6)	3 (7.0)	6 (9.8)	0.584
Creatinine >1.5 mg/dL	31 (29.2)	5 (11.6)	8 (12.9)	0.010
Hemoglobin <10.0 g/dL	44 (41.5)	15 (34.9)	20 (32.3)	0.454
Bone lytic lesions	55 (51.9)	24 (55.8)	35 (56.5)	0.983
Lactate dehydrogenase ≥ ULN, N (%)	26 (25.5)	5 (11.6)	6 (10.2)	0.024
Plasmacytoma, N (%)	25 (23.6)	7 (16.3)	8 (12.9)	0.206
ECOG PS, N (%)				<0.001
0	6 (5.8)	15 (35.7)	20 (33.3)	
1	31 (29.8)	20 (47.6)	25 (41.7)	
2	35 (33.7)	7 (16.7)	11 (18.3)	
3	27 (26.0)	0 (0.0)	4 (6.7)	
4	5 (4.8)	0 (0.0)	0 (0.0)	
ECOG PS >2, N (%)	32 (30.8)	0 (0.0)	4 (6.7)	<0.001
ISS, n (%) (*N* = 201)				0.853
I	20 (20.6)	11 (25.6)	16 (26.2)	
II	29 (29.9)	13 (30.2)	20 (32.8)	
III	48 (49.5)	19 (44.2)	25 (41.0)	
Treatment combinations, N (%)				<0.001
Chemotherapy based	84 (79.2)	5 (11.6)	5 (8.1)	
IMiD-based	2 (1.9)	13 (30.2)	11 (17.7)	
PI-based	20 (18.9)	19 (44.2)	26 (41.9)	
IMiD + PI-based	0 (0.0)	6 (14.0)	9 (14.5)	
Monoclonal antibody-based	0 (0.0)	0 (0)	11 (17.7)	
Lines of Therapy, N (%)				<0.001
1	51 (48.1)	9 (20.9)	19 (30.6)	
2	28 (26.4)	16 (37.2)	10 (16.1)	
≥3	27 (25.5)	18 (41.9)	33 (53.3)	

CG: control group; ECOG PS: East Cooperative Oncology Group Performance Status; EG: experimental group; IQR: interquartile range; IMiD: immunomodulatory drug; ISS: International Staging System; NCT: non-clinical trial; PI: proteasome inhibitor; ULN: upper limit of normal.

**Table 5:** Summary of response rates after clinical trial enrollment or not enrollment.

Response	No Trial Group	Control Group	Experimental Group	*p*-Value
Entire Time Period, *N* (%)	106	43	62	<0.001
CR	3 (2.8)	7 (16.3)	10 (16.1)	
PR	43 (40.6)	25 (58.1)	37 (59.7)	
SD or PD	60 (56.6)	11 (25.6)	15 (24.2)	
ORR	46 (43.4)	32 (74.4)	47 (75.8)	
2003–2007, N (%)	35	10	13	0.336
CR	1 (2.9)	2 (20)	0 (0.0)	
PR	16 (45.7)	5 (50.0)	8 (61.5)	
SD or PD	18 (51.4)	3 (30.0)	5 (38.5)	
ORR	17 (48.6)	7 (70)	8 (61.5)	
2008–2012, N (%)	37	10	24	0.223
CR	1 (2.7)	1 (10.0)	3 (12.5)	
PR	14 (37.8)	7 (70.0)	15 (62.6)	
SD or PD	22 (59.5)	2 (20.0)	6 (24.9)	
ORR	15 (40.5)	8 (80)	18 (75.1)	
2013–2017, N (%)	34	23	25	0.002
CR	1 (2.9)	4 (17.4)	7 (28.0)	
PR	13 (38.2)	13 (56.5)	14 (56.0)	
SD or PD	20 (58.9)	6 (26.1)	4 (16.0)	
ORR	14 (41.1)	17 (73.9)	21 (84.0)	

CR: complete response; ORR: overall response rate; PD: progressive disease; PR: partial response; SD: stable disease.

**Table 6:** Univariate and multivariate logistic regression model for ORR.

Variable	No. of Events/Total No.		Univariate			Multivariate		
	Not Enrolled	Enrolled	OR	95% CI	*p*	OR	95% CI	*p*
Gender, Male	22/45	43/54	1.66	0.95–2.89	0.083	—	—	—
Age, >75 years	23/64	20/31	0.34	0.19–0.61	<0.001	0.43	0.23–0.80	0.008
Year of treatment								
2008–2012	15/37	27/34	1.11	0.55–2.24	0.772	—	—	—
2013–2017	14/34	38/48	1.41	0.71–2.79	0.331	—	—	—
Heavy chain, non-IgG	22/47	42/53	1.46	0.84–2.53	0.183	—	—	—
Light chain, lambda	19/44	33/40	1.11	0.63–1.96	0.736	—	—	—
CRAB								
Calcium >11.0 mg/dL	4/13	7/9	0.65	0.27–1.58	0.344	—	—	—
Creatinine >1.5 mg/dL	10/31	8/13	0.36	0.18–0.73	0.004	0.42	0.20–0.89	0.021
Hemoglobin <10.0 g/dL	15/44	25/35	0.59	0.33–1.04	0.072	—	—	—
Bone lytic lesions	24/55	43/59	0.85	0.48–1.50	0.575	—	—	—
Lactate dehydrogenase	11/26	9/11	0.77	0.38–1.57	0.472	—	—	—
Plasmacytoma	11/25	12/15	0.92	0.46–1.84	0.808	—	—	—
ECOG PS, >2	9/32	2/4	0.23	0.11–0.50	<0.001	—	—	—
ISS								
II	18/29	29/33	1.94	0.85–4.45	0.125	—	—	—
III	14/48	31/44	0.59	0.29–1.22	0.152	—	—	—
Clinical trial enrollment	46/106	79/105	3.96	2.20–7.12	<0.001	3.39	1.78–6.44	0.0002

Variables with a *p* ≤ 0.1 were included in the multivariate model. CI: Confidence interval; ECOG PS: East Cooperative Oncology Group Performance Status; ISS: International Staging System; OR: Odds ratio; ORR: overall response rate.

**Table 7:** Univariate and multivariate Cox proportional hazard regression with time-dependent covariate model for PFS.

Variable	No. of Events/Total No.		Univariate			Multivariate		
	Not Enrolled	Enrolled	HR	95% CI	*p*	HR	95% CI	*p*
Gender, Male	45/45	48/54	1.19	0.90–1.57	0.226	—	—	—
Age, >75 years	64/64	31/31	1.18	0.86–1.63	0.313	—	—	—
Year of treatment								
2008–2012	37/37	34/34	0.99	0.70–1.41	0.961	—	—	—
2013–2017	34/34	41/48	0.78	0.55–1.11	0.164	—	—	—
Heavy chain, non-IgG	46/47	50/53	1.28	0.97–1.69	0.088	—	—	—
Light chain, lambda	43/44	38/40	1.37	0.34–5.59	0.489	—	—	—
CRAB								
Calcium >11.0 mg/dL	13/13	8/9	1.77	1.12–2.79	0.014	1.71	1.06–2.76	0.029
Creatinine >1.5 mg/dL	30/31	12/13	1.65	1.17–2.32	0.004	1.61	1.12–2.32	0.011
Hemoglobin <10.0 g/dL	43/44	31/35	1.05	0.79–1.40	0.962	—	—	—
Bone lytic lesions	54/55	55/59	1.13	0.85–1.50	0.308	—	—	—
Lactate dehydrogenase	25/26	10/11	1.23	0.85–1.77	0.579	—	—	—
Plasmacytoma	25/25	15/15	1.56	1.10–2.21	0.021	1.72	1.19–2.48	0.004
ECOG PS, >2	32/32	3/4	1.52	1.03–2.23	0.034	—	—	—
ISS								
II	29/29	27/33	0.79	0.53–1.16	0.228	—	—	—
III	48/48	42/44	1.23	0.86–1.76	0.251	—	—	—
Clinical trial enrollment	105/106	97/105	0.67	0.51–0.89	0.005	0.70	0.52–0.94	0.019

Variables with a *p* ≤ 0.1 were included in the multivariate model. The backward stepwise method was used to identify factors associated with PFS. CI: Confidence interval; ECOG PS: East Cooperative Oncology Group Performance Status; HR: hazard ratio; ISS: International Staging System.

**Table 8:** Univariate and multivariate Cox proportional hazard regression with time-dependent covariate model for OS.

Variable	No. of Events/Total No.		Univariate			Multivariate		
	Not Enrolled	Enrolled	HR	95% CI	*p*	HR	95% CI	*p*
Gender, Male	44/45	39/54	1.25	0.93–1.69	0.147	—	—	—
Age, >75 years	62/64	27/31	2.16	1.58–2.95	<0.001	1.69	1.19–2.38	0.003
Year of treatment								
2008–2012	36/37	29/34	0.88	0.61–1.26	0.485	—	—	—
2013–2017	30/34	23/48	0.70	0.48–1.03	0.069	—	—	—
Heavy chain, non-IgG	46/47	50/53	1.17	0.87–1.58	0.314	—	—	—
Light chain, lambda	45/44	38/40	0.87	0.64–1.19	0.381	—	—	—
CRAB								
Calcium >11.0 mg/dL	13/13	6/9	1.84	1.14–2.97	0.013	—	—	—
Creatinine >1.5 mg/dL	30/31	12/13	2.14	1.51–3.04	<0.001	1.60	1.03–2.49	0.040
Hemoglobin <10.0 g/dL	40/44	25/35	1.07	0.79–1.46	0.665	—	—	—
Bone lytic lesions	48/55	41/59	0.96	0.71–1.31	0.797	—	—	
Lactate dehydrogenase	23/26	8/11	1.34	0.90–1.98	0.148	—	—	—
Plasmacytoma	23/25	12/15	1.50	1.03–2.17	0.035	2.28	1.46–3.56	<0.001
ECOG PS, >2	32/32	3/4	2.98	2.03–4.38	<0.001	1.62	1.04–2.49	0.041
ISS								
II	29/29	17/33	1.25	0.80–1.95	0.337	—	—	—
III	4/48	36/44	2.04	1.36–3.05	<0.001	2.07	1.25–3.44	0.005
Clinical trial enrollment	99/106	72/105	0.44	0.32–0.60	<0.001	0.60	0.42–0.86	0.003

Variables with a *p* ≤ 0.1 were included in the multivariate model. The backward stepwise method was used to identify factors associated with OS. CI: Confidence interval; ECOG PS: East Cooperative Oncology Group Performance Status; HR: hazard ratio; ISS: International Staging System.

## Data Availability

The data presented in this study are available upon request from the corresponding author.

## Supplementary Materials

The following supporting information can be downloaded at: https://www.mdpi.com/article/10.3390/cancers15215261/s1, Figure S1: Study flow chart; Figure S2: Number of patients enrolled in a clinical trial; Figure S3: Time elapsed between diagnosis and initial treatment; Table S1: 3-year progression-free survival estimation during the three periods analyzed; Table S2: 5-year overall survival estimation during the three periods analyzed.
