# Supplementary material for: Outcomes of Patients with Newly Diagnosed Transplant-Ineligible Multiple Myeloma According to Clinical Trials Enrollment: Experience of a Single Institution
Source: Cancers (Basel). 2023 Nov 2;15(21):5261. doi: 10.3390/cancers15215261 (PMC10649258; doi:10.3390/cancers15215261)

Supplementary Materials.

Supplementary Table S1. 3-year Progression-Free Survival estimation during the three periods analyzed

Period	Not Enrolled in CT<br>3y-PFS (%)<br>95% CI<br>Events / Total	Control Group<br>3y-PFS (%)<br>95% CI<br>Events / Total	Experimental Group<br>3y-PFS (%)<br>95% CI<br>Events / Total
**All patients**	17.3<br>10.8-25.2<br>87/106	18.6<br>8.7-31.4<br>35/43	25.8<br>15.7-37.1<br>46/62
**2003-2007**	9.1<br>2.3-21.7<br>31/35	14.5<br>7.1-57.8<br>7/10	7.7<br>0.5-29.2<br>12/13
**2008-2012**	21.6<br>10.2-35.8<br>29/37	20.0<br>3.1-47.5<br>8/10	16.7<br>5.2-33.7<br>20/24
**2013-2017**	20.6<br>9.1-35.3<br>27/34	13.0<br>3.3-29.7<br>20/23	44.0<br>24.5-61.9<br>14/25

CI: confidence interval; CT: clinical trial; PFS: progression-free survival.

Supplementary Table S2. 5-year Overall Survival estimation during the three periods analyzed

Period	Not Enrolled in CT<br>5y-OS (%)<br>95% CI<br>Events / Total	Control Group<br>5y-OS (%)<br>95% CI<br>Events / Total	Experimental Group<br>5y-OS (%)<br>95% CI<br>Events / Total
**All patients**	25.3<br>17.4-33.9<br>78/106	60.3<br>44.1-73.1<br>17/43	54.7<br>41.6-66.1<br>28/62
**2003-2007**	21.9<br>9.7-37.2<br>26/35	60.0<br>15.5-25.3<br>4/10	38.5<br>14.1-62.8<br>8/13
**2008-2012**	32.4<br>18.2-47.5<br>25/37	60.0<br>25.3-82.7<br>4/10	50<br>29.1-67.8<br>12/24
**2013-2017**	20.6<br>9.1-35.3<br>27/34	60.6<br>37.8-77.2<br>9/23	68.0<br>46.1-82.5<br>8/25

CI: confidence interval; CT: clinical trial; OS: overall survival.

Supplementary Figure S1. Study flow chart. Patients were excluded if they were eligible to autologous stem cell transplantation and if there were no clinical trial available at the time of starting first-line treatment.

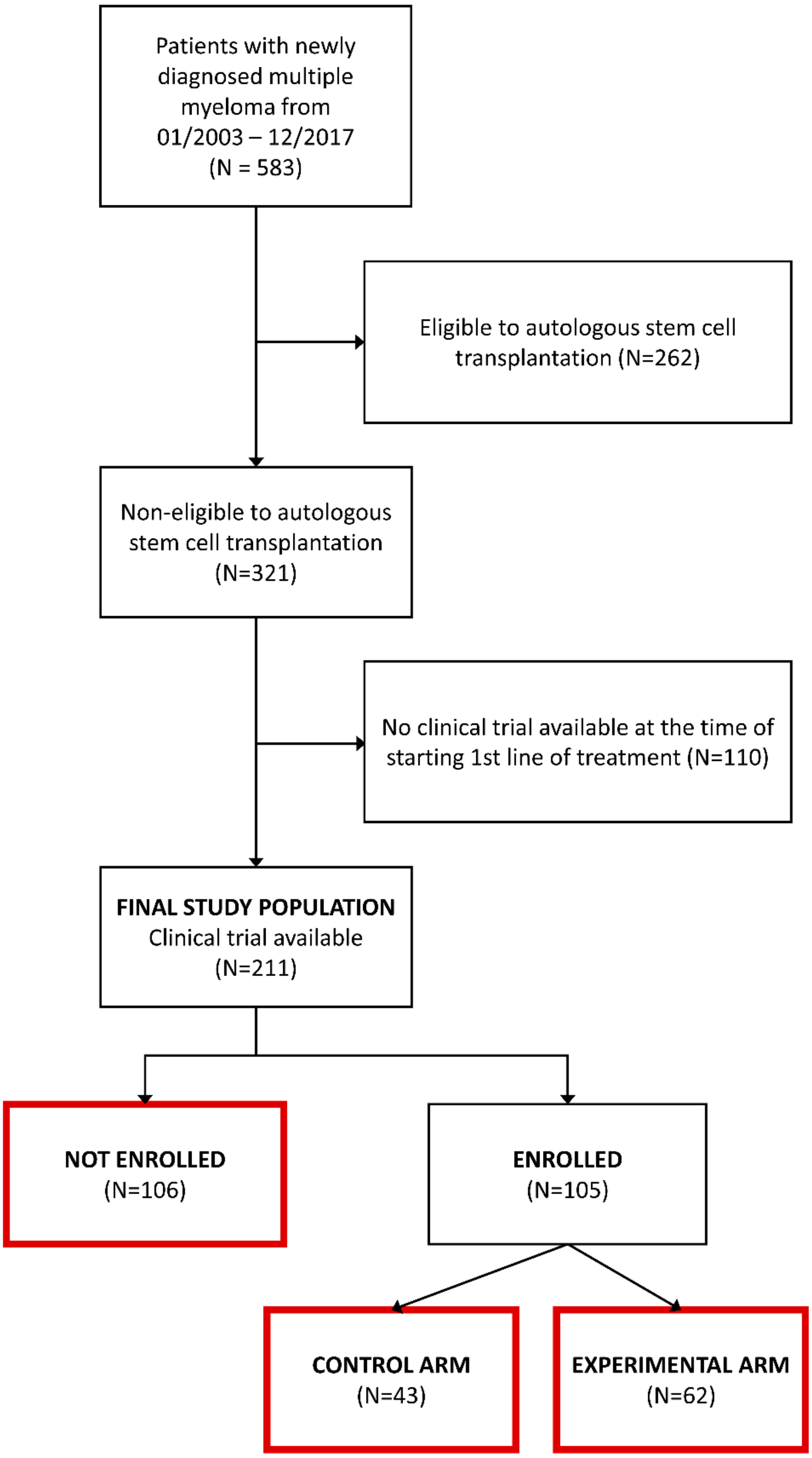

Supplementary Figure S2. Number of patients enrolled in a clinical trial. The bars represent de number of patients included (control and experimental arms) and not included in clinical trials during 3 calendar periods (2003-2007, 2008-2012, and 2013-2017).

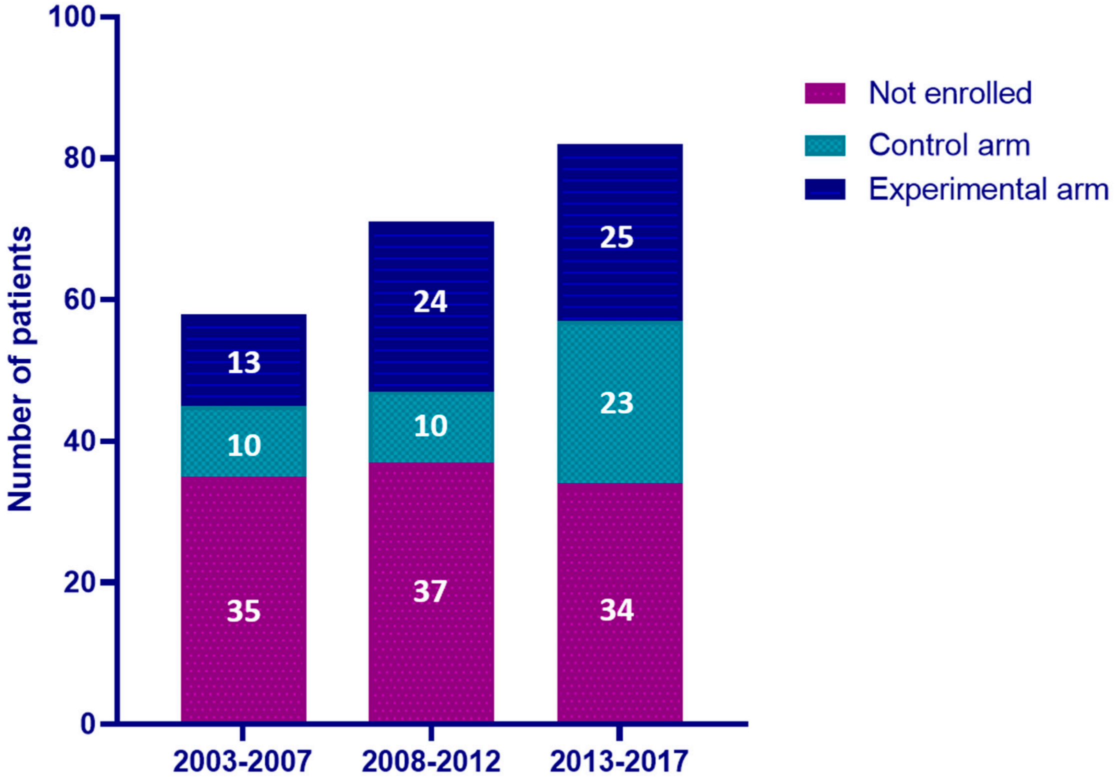

Supplementary Figure S3. Time elapsed between diagnosis and initial treatment

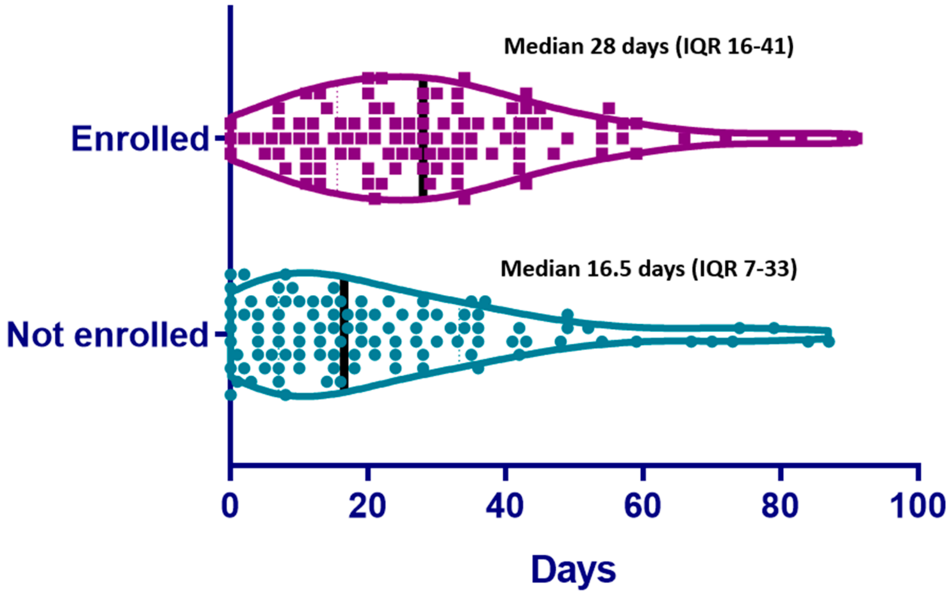

Supplement: Supplementary file 1 [file cancers-15-05261-s001.zip › cancers-2625243-supplementary.pdf]
